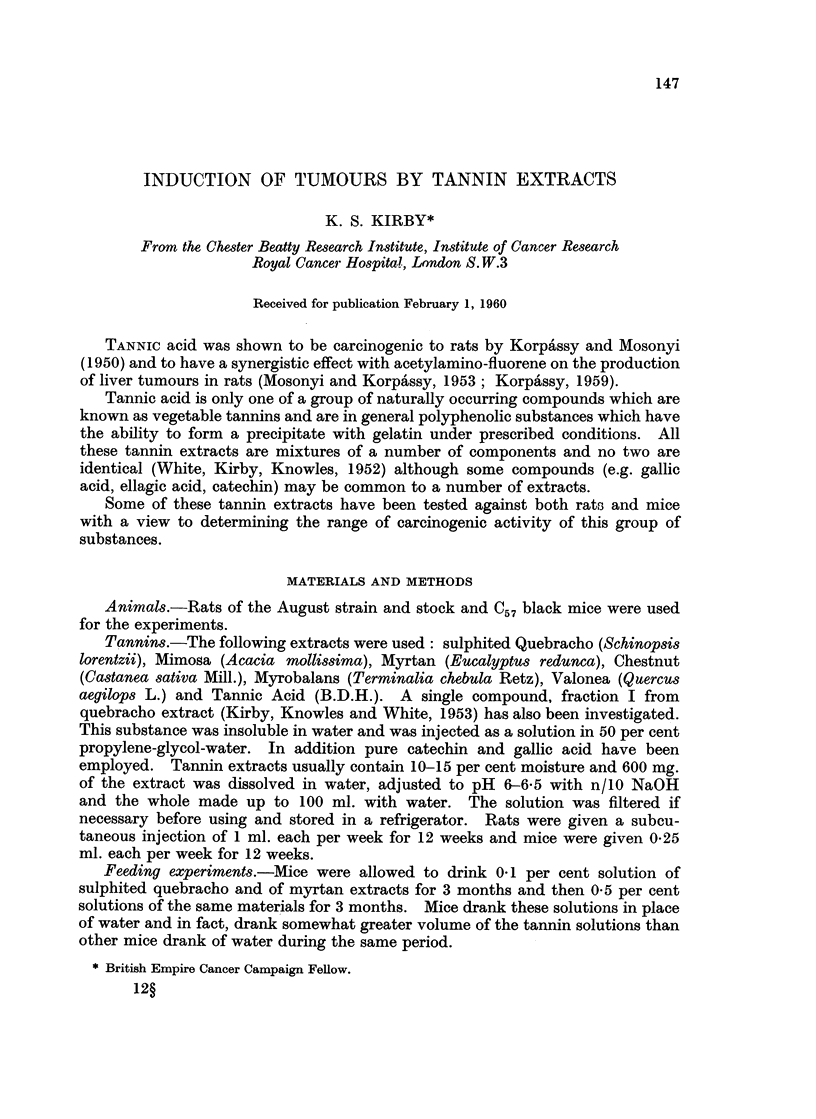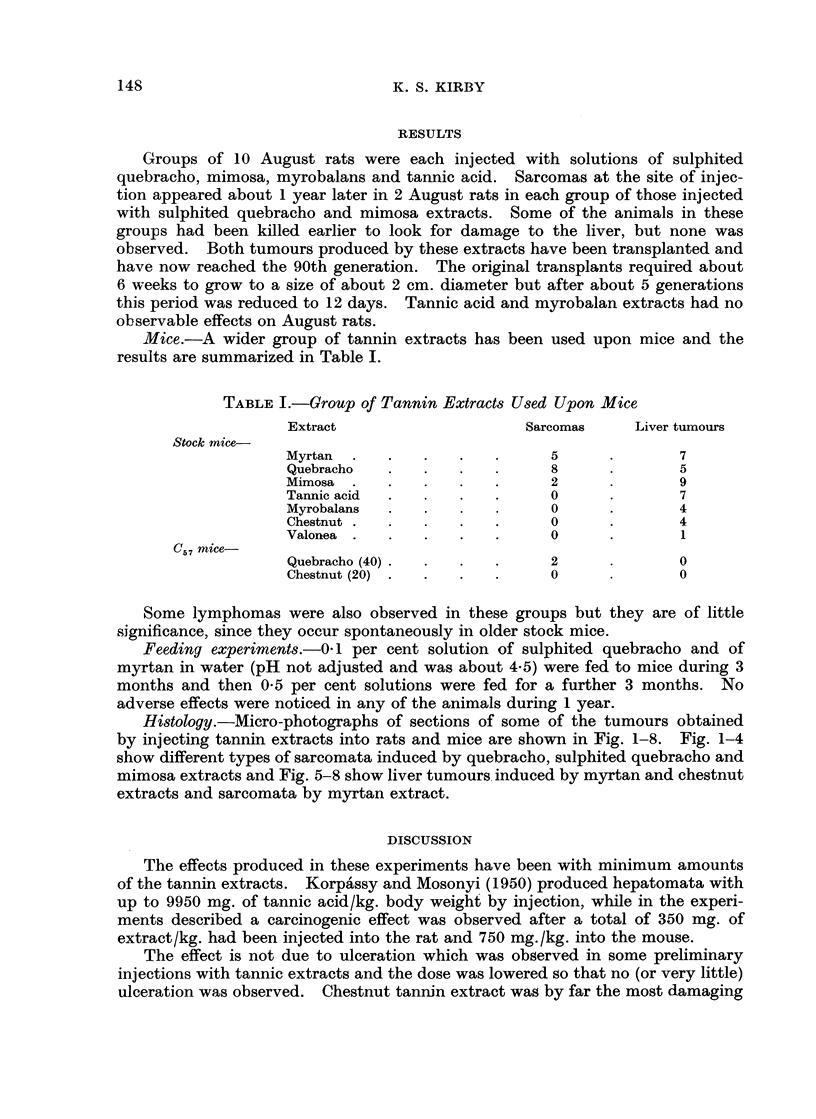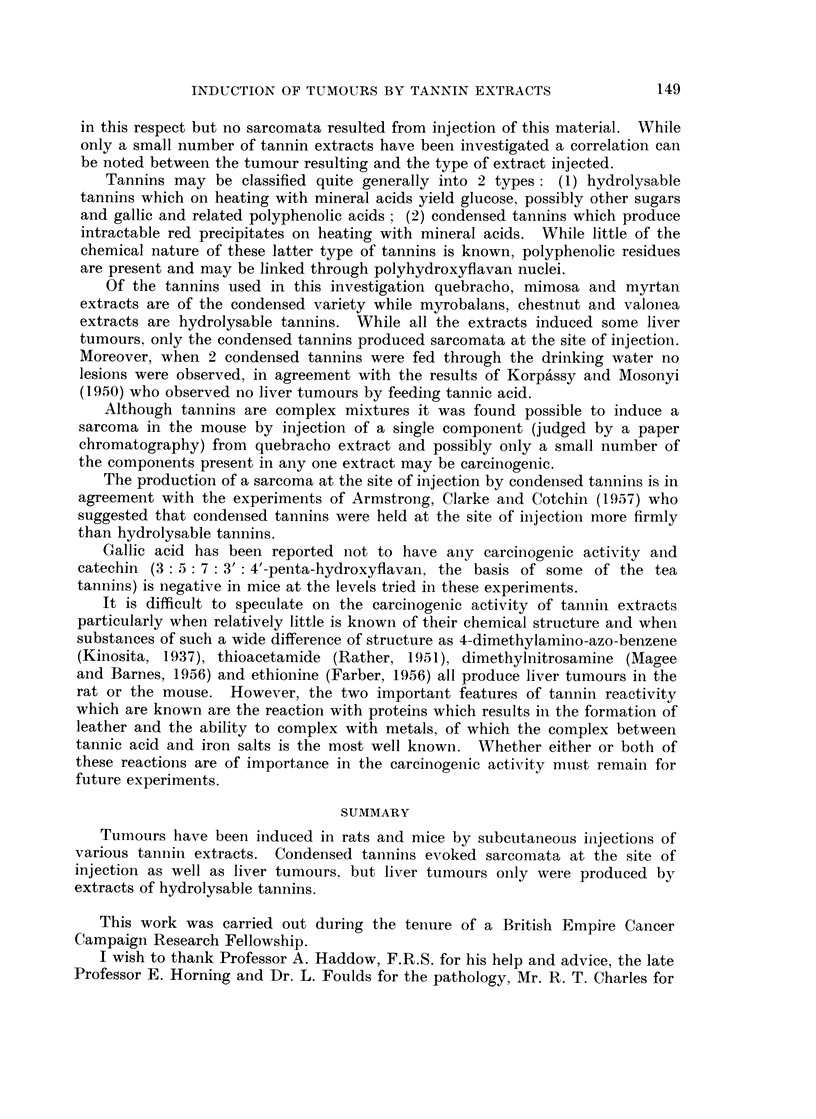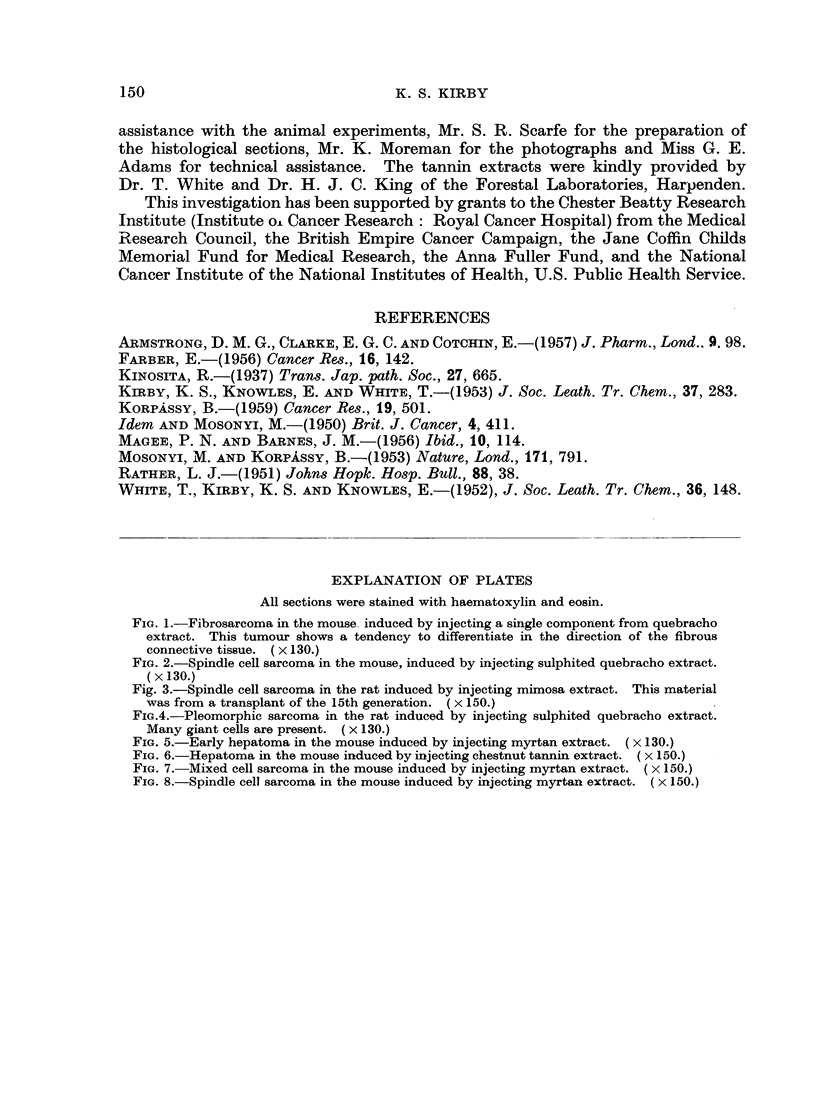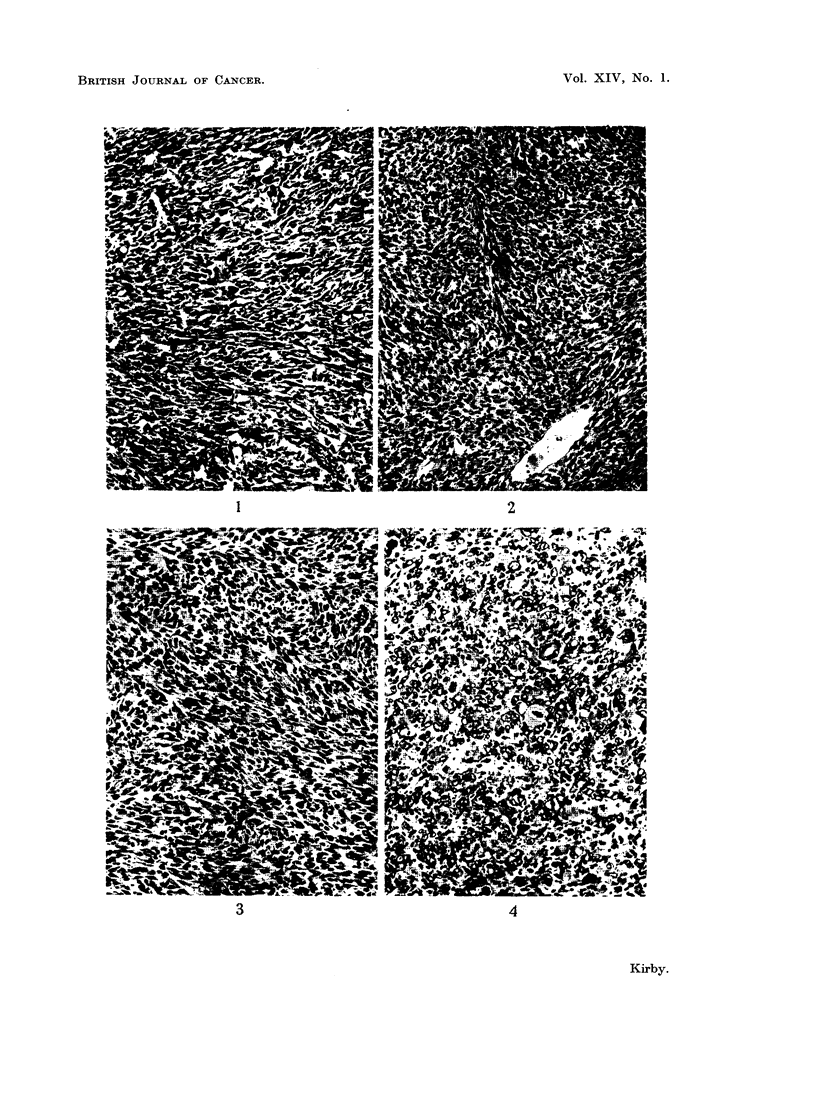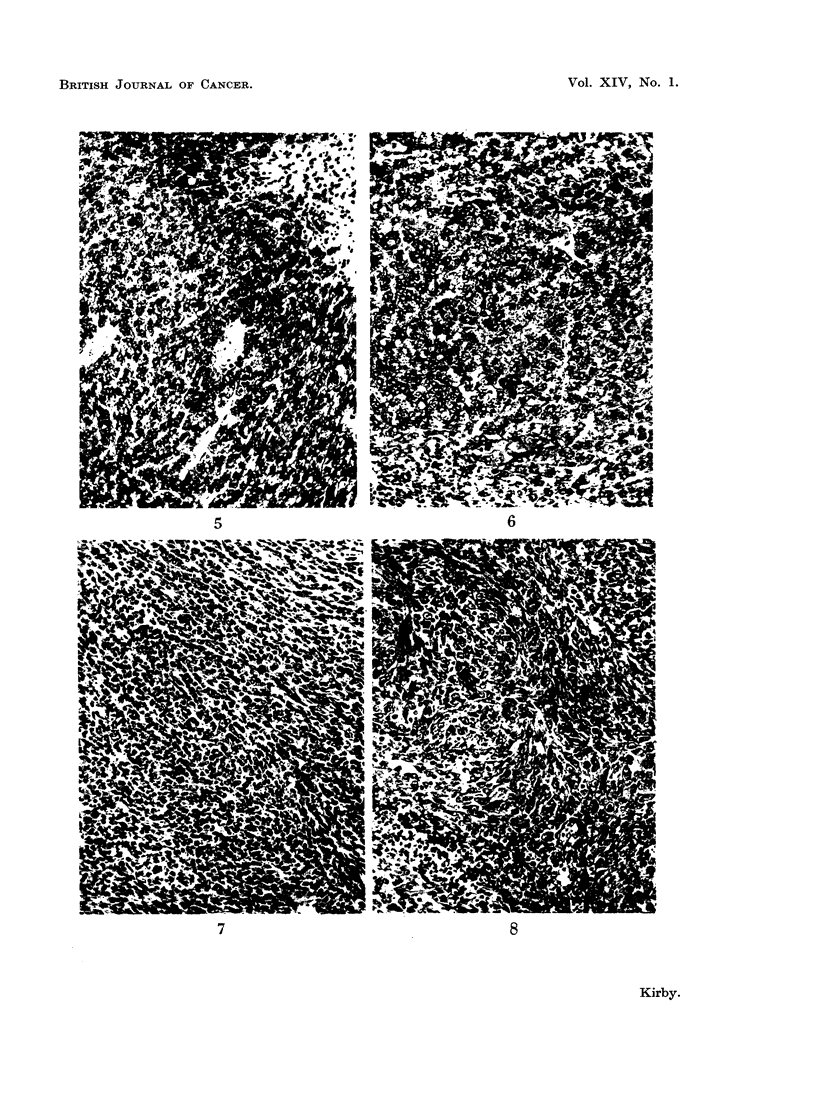# Induction of Tumours by Tannin Extracts

**DOI:** 10.1038/bjc.1960.17

**Published:** 1960-03

**Authors:** K. S. Kirby

## Abstract

**Images:**


### INDUCTION OF TUMOURS BY TANNIN EXTRACTS

K. S. KIRBY*

*From the Chester Beatty Research Institute, Institute of Cancer Research Royal Cancer Hospital, London S.W.3*

Received for publication February 1, 1960

TANNIC acid was shown to be carcinogenic to rats by Korpássy and Mosonyi (1950) and to have a synergistic effect with acetylamino-fluorene on the production of liver tumours in rats (Mosonyi and Korpássy, 1953 ; Korpássy, 1959).

Tannic acid is only one of a group of naturally occurring compounds which are known as vegetable tannins and are in general polyphenolic substances which have the ability to form a precipitate with gelatin under prescribed conditions. All these tannin extracts are mixtures of a number of components and no two are identical (White, Kirby, Knowles, 1952) although some compounds (e.g. gallic acid, ellagic acid, catechin) may be common to a number of extracts.

Some of these tannin extracts have been tested against both rats and mice with a view to determining the range of carcinogenic activity of this group of substances.

### MATERIALS AND METHODS

*Animals.*—Rats of the August strain and stock and $C_{57}$ black mice were used for the experiments.

*Tannins.*—The following extracts were used : sulphited Quebracho (*Schinopsis lorentzii*), Mimosa (*Acacia mollissima*), Myrtan (*Eucalyptus redunca*), Chestnut (*Castanea sativa* Mill.), Myrobalans (*Terminalia chebula* Retz), Valonea (*Quercus aegilops* L.) and Tannic Acid (B.D.H.). A single compound, fraction I from quebracho extract (Kirby, Knowles and White, 1953) has also been investigated. This substance was insoluble in water and was injected as a solution in 50 per cent propylene-glycol-water. In addition pure catechin and gallic acid have been employed. Tannin extracts usually contain 10–15 per cent moisture and 600 mg. of the extract was dissolved in water, adjusted to pH 6–6·5 with n/10 NaOH and the whole made up to 100 ml. with water. The solution was filtered if necessary before using and stored in a refrigerator. Rats were given a subcutaneous injection of 1 ml. each per week for 12 weeks and mice were given 0·25 ml. each per week for 12 weeks.

*Feeding experiments.*—Mice were allowed to drink 0·1 per cent solution of sulphited quebracho and of myrtan extracts for 3 months and then 0·5 per cent solutions of the same materials for 3 months. Mice drank these solutions in place of water and in fact, drank somewhat greater volume of the tannin solutions than other mice drank of water during the same period.

* British Empire Cancer Campaign Fellow.

### RESULTS

Groups of 10 August rats were each injected with solutions of sulphited quebracho, mimosa, myrobalans and tannic acid. Sarcomas at the site of injection appeared about 1 year later in 2 August rats in each group of those injected with sulphited quebracho and mimosa extracts. Some of the animals in these groups had been killed earlier to look for damage to the liver, but none was observed. Both tumours produced by these extracts have been transplanted and have now reached the 90th generation. The original transplants required about 6 weeks to grow to a size of about 2 cm. diameter but after about 5 generations this period was reduced to 12 days. Tannic acid and myrobalan extracts had no observable effects on August rats.

*Mice.*—A wider group of tannin extracts has been used upon mice and the results are summarized in Table I.

TABLE I.—*Group of Tannin Extracts Used Upon Mice*

| | Extract | Sarcomas | Liver tumours |
|---|---|---|---|
| *Stock mice*— | | | |
| | Myrtan | 5 | 7 |
| | Quebracho | 8 | 5 |
| | Mimosa | 2 | 9 |
| | Tannic acid | 0 | 7 |
| | Myrobalans | 0 | 4 |
| | Chestnut | 0 | 4 |
| | Valonea | 0 | 1 |
| $C_{57}$ *mice*— | | | |
| | Quebracho (40) | 2 | 0 |
| | Chestnut (20) | 0 | 0 |

Some lymphomas were also observed in these groups but they are of little significance, since they occur spontaneously in older stock mice.

*Feeding experiments.*—0·1 per cent solution of sulphited quebracho and of myrtan in water (pH not adjusted and was about 4·5) were fed to mice during 3 months and then 0·5 per cent solutions were fed for a further 3 months. No adverse effects were noticed in any of the animals during 1 year.

*Histology.*—Micro-photographs of sections of some of the tumours obtained by injecting tannin extracts into rats and mice are shown in Fig. 1–8. Fig. 1–4 show different types of sarcomata induced by quebracho, sulphited quebracho and mimosa extracts and Fig. 5–8 show liver tumours induced by myrtan and chestnut extracts and sarcomata by myrtan extract.

### DISCUSSION

The effects produced in these experiments have been with minimum amounts of the tannin extracts. Korpássy and Mosonyi (1950) produced hepatomata with up to 9950 mg. of tannic acid/kg. body weight by injection, while in the experiments described a carcinogenic effect was observed after a total of 350 mg. of extract/kg. had been injected into the rat and 750 mg./kg. into the mouse.

The effect is not due to ulceration which was observed in some preliminary injections with tannic extracts and the dose was lowered so that no (or very little) ulceration was observed. Chestnut tannin extract was by far the most damaging in this respect but no sarcomata resulted from injection of this material. While only a small number of tannin extracts have been investigated a correlation can be noted between the tumour resulting and the type of extract injected.

Tannins may be classified quite generally into 2 types : (1) hydrolysable tannins which on heating with mineral acids yield glucose, possibly other sugars and gallic and related polyphenolic acids ; (2) condensed tannins which produce intractable red precipitates on heating with mineral acids. While little of the chemical nature of these latter type of tannins is known, polyphenolic residues are present and may be linked through polyhydroxyflavan nuclei.

Of the tannins used in this investigation quebracho, mimosa and myrtan extracts are of the condensed variety while myrobalans, chestnut and valonea extracts are hydrolysable tannins. While all the extracts induced some liver tumours, only the condensed tannins produced sarcomata at the site of injection. Moreover, when 2 condensed tannins were fed through the drinking water no lesions were observed, in agreement with the results of Korpássy and Mosonyi (1950) who observed no liver tumours by feeding tannic acid.

Although tannins are complex mixtures it was found possible to induce a sarcoma in the mouse by injection of a single component (judged by a paper chromatography) from quebracho extract and possibly only a small number of the components present in any one extract may be carcinogenic.

The production of a sarcoma at the site of injection by condensed tannins is in agreement with the experiments of Armstrong, Clarke and Cotchin (1957) who suggested that condensed tannins were held at the site of injection more firmly than hydrolysable tannins.

Gallic acid has been reported not to have any carcinogenic activity and catechin (3 : 5 : 7 : 3′ : 4′-penta-hydroxyflavan, the basis of some of the tea tannins) is negative in mice at the levels tried in these experiments.

It is difficult to speculate on the carcinogenic activity of tannin extracts particularly when relatively little is known of their chemical structure and when substances of such a wide difference of structure as 4-dimethylamino-azo-benzene (Kinosita, 1937), thioacetamide (Rather, 1951), dimethylnitrosamine (Magee and Barnes, 1956) and ethionine (Farber, 1956) all produce liver tumours in the rat or the mouse. However, the two important features of tannin reactivity which are known are the reaction with proteins which results in the formation of leather and the ability to complex with metals, of which the complex between tannic acid and iron salts is the most well known. Whether either or both of these reactions are of importance in the carcinogenic activity must remain for future experiments.

### SUMMARY

Tumours have been induced in rats and mice by subcutaneous injections of various tannin extracts. Condensed tannins evoked sarcomata at the site of injection as well as liver tumours, but liver tumours only were produced by extracts of hydrolysable tannins.

This work was carried out during the tenure of a British Empire Cancer Campaign Research Fellowship.

I wish to thank Professor A. Haddow, F.R.S. for his help and advice, the late Professor E. Horning and Dr. L. Foulds for the pathology, Mr. R. T. Charles for assistance with the animal experiments, Mr. S. R. Scarfe for the preparation of the histological sections, Mr. K. Moreman for the photographs and Miss G. E. Adams for technical assistance. The tannin extracts were kindly provided by Dr. T. White and Dr. H. J. C. King of the Forestal Laboratories, Harpenden.

This investigation has been supported by grants to the Chester Beatty Research Institute (Institute oı Cancer Research : Royal Cancer Hospital) from the Medical Research Council, the British Empire Cancer Campaign, the Jane Coffin Childs Memorial Fund for Medical Research, the Anna Fuller Fund, and the National Cancer Institute of the National Institutes of Health, U.S. Public Health Service.

### REFERENCES

ARMSTRONG, D. M. G., CLARKE, E. G. C. AND COTCHIN, E.—(1957) *J. Pharm., Lond.*, **9**, 98.
FARBER, E.—(1956) *Cancer Res.*, **16**, 142.
KINOSITA, R.—(1937) *Trans. Jap. path. Soc.*, **27**, 665.
KIRBY, K. S., KNOWLES, E. AND WHITE, T.—(1953) *J. Soc. Leath. Tr. Chem.*, **37**, 283.
KORPÁSSY, B.—(1959) *Cancer Res.*, **19**, 501.
*Idem* AND MOSONYI, M.—(1950) *Brit. J. Cancer*, **4**, 411.
MAGEE, P. N. AND BARNES, J. M.—(1956) *Ibid.*, **10**, 114.
MOSONYI, M. AND KORPÁSSY, B.—(1953) *Nature, Lond.*, **171**, 791.
RATHER, L. J.—(1951) *Johns Hopk. Hosp. Bull.*, **88**, 38.
WHITE, T., KIRBY, K. S. AND KNOWLES, E.—(1952), *J. Soc. Leath. Tr. Chem.*, **36**, 148.

---

### EXPLANATION OF PLATES

All sections were stained with haematoxylin and eosin.

FIG. 1.—Fibrosarcoma in the mouse induced by injecting a single component from quebracho extract. This tumour shows a tendency to differentiate in the direction of the fibrous connective tissue. (×130.)

FIG. 2.—Spindle cell sarcoma in the mouse, induced by injecting sulphited quebracho extract. (×130.)

Fig. 3.—Spindle cell sarcoma in the rat induced by injecting mimosa extract. This material was from a transplant of the 15th generation. (×150.)

FIG.4.—Pleomorphic sarcoma in the rat induced by injecting sulphited quebracho extract. Many giant cells are present. (×130.)

FIG. 5.—Early hepatoma in the mouse induced by injecting myrtan extract. (×130.)

FIG. 6.—Hepatoma in the mouse induced by injecting chestnut tannin extract. (×150.)

FIG. 7.—Mixed cell sarcoma in the mouse induced by injecting myrtan extract. (×150.)

FIG. 8.—Spindle cell sarcoma in the mouse induced by injecting myrtan extract. (×150.)